# Clinical Breast Examination Uptake among Reproductive Aged Women in Zambia: Evidence from the 2024 Zambia Demographic and Health Survey

**DOI:** 10.21203/rs.3.rs-8380869/v1

**Published:** 2026-01-08

**Authors:** Penias Tembo, David Owiredu, Edson Chipalo, Charles Michelo, James R. Hébert

**Affiliations:** University of South Carolina; University of South Carolina; University of Cincinnati; Nkwazi Research University; University of South Carolina

**Keywords:** Prevalence, Breast Cancer Screening, Clinical Breast Examination, Females of Reproductive Age, Zambia Demographic and Health Survey

## Abstract

**Introduction:**

Breast cancer remains a major cause of mortality among women in sub-Saharan Africa driven, in part, by low screening coverage. We assessed the prevalence and determinants of clinical breast examination (CBE) uptake among reproductive-aged women in Zambia.

**Methods:**

This cross-sectional study utilized data from 13,876 women aged 15–49 years who participated in the 2024 Zambia Demographic and Health Survey (ZDHS). The outcome variable was derived from participants’ response to ‘ever having had a breast examination by a health care provider.’ Descriptive statistics and survey-weighted logistic regression analyses were conducted in Stata, with all estimates adjusted to account for the complex survey design used in the ZDHS. Adjusted odds ratios (AORs) and 95% confidence intervals (CIs) were reported.

**Results:**

Overall, 13.3% (n = 1,845) of women reported ever receiving CBE. Among those screened, the majority were aged 30–39 years (35.4%), resided in urban areas (56.5%), had secondary level education (42.7%) and belonged to the richest wealth quintile (30.5%). Furthermore, only 17.8% of screened women had health insurance, and two-thirds (66.2%) had previously undergone cervical cancer screening. Higher education (AOR = 1.58; 95% CI: 1.08–2.32) and health insurance coverage (AOR = 1.30; 95% CI: 1.04–1.62) were positively associated with CBE uptake. Women who had visited a health facility in the previous 12 months (AOR = 1.38; 95% CI: 1.18–1.61) and those ever screened for cervical cancer (AOR = 5.95; 95% CI: 5.18–6.84) had significantly greater odds of receiving CBE, while women living with HIV were less likely to be screened (AOR = 0.82; 95% CI: 0.69–0.98). Regional variation was observed with women in Central (AOR = 1.43; 95% CI: 1.10–1.88), Copperbelt (AOR = 1.42; 95% CI: 1.12–1.78), Luapula (AOR = 1.94; 95% CI: 1.39–2.72), and Northwestern (AOR = 1.33; 95% CI: 1.01–1.77) provinces having higher odds of CBE compared with those in Lusaka, while women in Eastern Province were less likely to receive CBE (AOR = 0.68; 95% CI: 0.49–0.95).

**Conclusion:**

There is low utilization of CBE services among women aged 15–49 years in Zambia. Strategies which expand access and utilization of breast cancer screening services are warranted.

## Introduction

Globally, breast cancer is the most frequently diagnosed cancer in women and the leading cause of cancer-related mortality in females, with an estimated 2.3 million new cases and 660,000 deaths reported in 2022.^1^ While breast cancer-specific mortality rates have declined in many high-income countries due to advances in early detection and treatment, sub-Saharan Africa continues to experience disproportionately high mortality rates due to late stage diagnoses and inadequate health infrastructure.^1–3^ In Zambia, one study reported the three-year survival following a diagnosis of breast cancer is estimated to be between 44% to 47%, which reflects significant challenges in the management continuum.^4^ This issue has far reaching intergenerational consequences, with evidence showing that for every 100 breast cancer deaths in sub-Saharan Africa, approximately 210 children lose their mothers, and the burden is especially high in Zambia, where about 247 maternal orphans result from every 100 deaths.^5^ Indeed, there is a critical need to address this high burden of mortality attributed to the disease.

Early detection through screening is critical for improving breast cancer survival outcomes. Screening modalities for breast cancer include ultrasound of the breast, mammography, clinical breast cancer examinations (CBE) and breast cancer self-examinations (BSE). The World Health Organization recommends population-based mammography as a screening modality for females aged 50–69 years of age in well-resourced settings.^6^ However, in low resource constrained lands where diagnostic capacity is limited, and health systems are weak, CBE has been suggested as an alternative.^6^ A systematic review which explored the effectiveness of CBE as a stand-alone screening modality reported its merits for consideration among health systems and service planners in Low- and Middle-Income Countries where a national screening program based on mammography would be prohibitively expensive.^7^ This is especially important given that the World Health Organization’s 2024 assessment of breast cancer control capacities in the African region found that only 5 out of 47 countries had established organized screening programs, leaving the vast majority to rely on opportunistic approaches.^8^

Despite the growing recognition of screening in breast cancer control, Msadabwe et al. reported that research in the cancer care pathway in Zambia has largely focused on diagnostics, with less than 3% focused on preventive efforts.^9^ A consultative meeting on breast cancer control in Zambia highlighted the need for greater investment in local research and needs assessments to develop effective context specific interventions which drive the direction of national programs.^10^ The national guidelines for early diagnosis of breast cancer in Zambia recommend that CBE be offered to all women with a breast health concern regardless of their age.^11^ In light of these recommendations, understanding the factors that influence CBE uptake among women nationwide is essential. Currently, based on our knowledge, no nationally representative study has explored the socio-demographic determinants of breast cancer screening uptake in Zambia. A recent systematic review (2024) involving 174 population-based studies did not report any study on breast cancer screening uptake in Zambia.^12^ This study, therefore, aims to examine the national prevalence and explore the determinants of breast cancer screening uptake among women of reproductive age in Zambia.

## Methods

### Study Design

This cross-sectional study utilized data from the 2024 Zambia Demographic and Health Survey (ZDHS), a nationally representative survey.^13^ The survey’s design and implementation procedures have been reported elsewhere.^13^ Briefly, the 2024 ZDHS employed a two-stage, stratified sampling design using the 2022 Census as its sampling frame, selecting 545 enumeration areas proportionally across 20 urban-rural strata. From each enumeration area, 25 households were systematically chosen, yielding a nationally representative sample of 13,625 households. Data was collected between January and July 2024.

#### Study Setting:

Zambia is a landlocked country located in Southern Africa. The country is bordered by The Democratic Republic of Congo to the north, Tanzania to the north-east, Malawi to the east, Mozambique, Zimbabwe, Botswana and Namibia to the South, and Angola to the west.^14^ Administratively, it is divided into ten provinces: Central, Copperbelt, Eastern, Luapula, Lusaka, Muchinga, Northern, North Western, Southern and Western provinces.^13^ According to the Zambia Statistics Agency’s 2022 Census report, the country has a population of about 19.6 million people, of whom approximately 10 million are female.^14^

### Outcome Variable

Breast cancer screening uptake was assessed via self-report. Women were asked whether a medical professional had examined their breasts to screen for cancer.^13^ This could involve a physical clinical breast examination where the provider manually checked for lumps or abnormalities, or the use of imaging tools like mammography to view breast tissue. Women aged 15–49 years who were unsure whether they had received a breast examination were excluded from the analysis (N = 50). In this study, we refer to this as CBE. This variable was coded as a binary outcome “0 = No” and “1 = Yes”.

### Predictor Variables

Predictor variables were selected *a priori* based on their known association with breast cancer screening uptake from previous studies.^12,15,16^ These variables included age, residence, region, education, wealth status, employment status, health insurance coverage, media exposure, marital status, age at menarche, age at first sex, parity, HIV status, self-reported health status, distance to a health facility, and previous screening for cervical cancer. Coding used for our predictor variables are presented in Supplementary Table 1.

### Statistical Analysis

Data analysis was conducted using Stata/SE 16.0. Frequencies with corresponding survey weighted percentages were presented to characterize the study population. The “svy” command which considers strata and primary sampling units was used to account for the complex survey design of the 2024 ZDHS. Bivariate and multivariable logistic regression analyses were conducted to determine the predictors of CBE uptake. Crude odds ratios and adjusted odds ratio with their respective 95% confidence intervals were presented. Multicollinearity among predictors was assessed using the variance inflation factor (VIF) after fitting the regression model. We assessed potential interaction effects between key predictors (i.e., (a) region × residence, (b) education × wealth, (c) education × health insurance, (d) health facility visit in the past 12 months × prior cervical cancer screening, (e) health facility visit in the past 12 months × HIV status) on CBE uptake by introducing multiplicative interaction terms into the multivariable logistic regression model. Interaction terms were evaluated using Wald tests, and interactions with p-values < 0.05 were considered statistically significant. Model adequacy and calibration were evaluated using the Hosmer-Lemeshow goodness of fit test with 10 groups.

## Results

A total of 13,876 women aged 15–49 years were included in the analysis, of whom 1,845 (13.3%) reported ever having had a clinical breast examination (Table 1). Screening uptake differed significantly across sociodemographic characteristics. Among women who had undergone CBE, the largest proportion were aged 30–39 years (35.4%), followed by women aged 40–49 years (25.5%), while adolescents aged 15–19 years represented the smallest proportion (7.9 %). More than half of screened women resided in urban areas (56.5%), compared with 51.0% urban residence in the national sample. Regionally, the highest proportions of screened women were from Lusaka (18.6%), Copperbelt (18.4%) and Central (13.7%) Provinces, whereas Muchinga Province contributed the smallest proportion (3.9%), followed by Western (5.6%) and Northwestern (5.7%). Furthermore, among women who were screened, those with secondary (42.7%) and primary education (35.8%) comprised the largest proportions. Notably, 15.8% of screened women had higher education which was approximately double the national prevalence of higher education (7.8%). With respect to wealth, the proportion of screened women was highest in the richest quintile (30.5%), decreasing progressively across the richer (22.3%) and middle (18.2%) quintiles, and reaching its lowest level among the poorest women (13.5%). Health insurance coverage among screened women (17.8%) was nearly two times higher than the overall national coverage (9.4%). Additionally, only 14.7% of women living with HIV reported receiving CBE, and CBE uptake was twice as high among women who had undergone cervical cancer screening (66.2%) compared with those who had not (33.8%).

Table 2 reports the factors associated with CBE uptake among women aged 15–49 years. In the adjusted analysis, age, residence, wealth status, employment status, media exposure, marital status, age at menarche, age at first sex, parity, self-reported health status, and perceived distance to a health facility were not associated with receiving CBE. However, regional differences were observed in CBE utilization. In comparison with Lusaka Province, women in Central (AOR = 1.43; 95% CI: 1.10–1.88), Copperbelt (AOR = 1.42; 95% CI: 1.12–1.78), Luapula (AOR = 1.94; 95% CI: 1.39–2.72), and Northwestern provinces (AOR = 1.33; 95% CI: 1.01–1.77) had higher odds of undergoing CBE, whereas women in Eastern Province had significantly lower odds (AOR = 0.68; 95% CI: 0.49–0.95). Education level was a statistically significant predictor of CBE uptake. Women with higher education had increased odds of CBE compared with those with no education (AOR = 1.58; 95% CI: 1.08–2.32), while primary and secondary education were not statistically significant predictors in the adjusted model. Health system factors demonstrated positive associations with CBE utilization. Women who had visited a health facility in the past 12 months had higher odds of receiving CBE (AOR = 1.38; 95% CI: 1.18–1.61), and those covered by health insurance were 30% more likely to undergo CBE than those without coverage (AOR = 1.30; 95% CI: 1.04–1.62). Women living with HIV had lower odds of CBE compared with HIV-negative women (AOR = 0.82; 95% CI: 0.69–0.98). Cervical cancer screening was the strongest predictor of CBE uptake. Women who had ever undergone cervical cancer screening had nearly six times higher odds of utilizing CBE services compared with those who had never been screened (AOR = 5.95; 95% CI: 5.18–6.84). No statistically significant interaction effects were observed between the selected potential predictors.

## Discussion

This nationally representative study is the first to report the prevalence of clinical breast examination (CBE) uptake among Zambian women of reproductive age and to identify its major socio-demographic determinants. The prevalence of CBE uptake among women aged 15–49 years was only 13.3%. Our results are lower than the 23.1% pooled prevalence of CBE from 68 population based studies in Low and Middle Income countries.^12^ However, this rate is similar to that reported across other sub-Saharan African countries.^15,16^ This emphasizes the low level of utilization of breast cancer screening services across many sub-Saharan African countries, including Zambia. This is especially concerning in the Zambian context given the evidence that most breast cancer cases, histologically determined to be estrogen/progesterone receptor positive, appear to occur below the age of 50 years.^17,18^

We identified higher education level as a significant predictor of breast cancer screening uptake, reinforcing evidence that education enhances women’s health literacy and ability to navigate preventive health services.^19^ Similar findings have been reported in other African countries.^20,21^ In many settings, higher education is closely correlated with increased socioeconomic status which, in turn, is associated with health service utilization. Although we found that women with tertiary education had significantly greater odds of being screened household wealth, on its own, was not a significant predictor of screening utilization. This suggests that the influence of education on screening uptake may operate more through enhanced health literacy, awareness of the benefits of early detection,^22^ and improved ability to navigate the health system than through financial means.

Women with health insurance coverage were more likely to be screened for breast cancer as compared to those without coverage. Similar results have been reported across the region.^23,24^ Insurance coverage reduces out-of-pocket costs, increases contact with the health system, and facilitates access to preventive services. In Zambia, the public sector is the main provider of health services and 90% of patients seek care in facilities owned and run by the government. Therefore, to strengthen financial protection and expand access, the National Health Insurance Scheme was established under the National Health Insurance Act No. 2 of 2018 and operationalized through Statutory Instrument No. 63 of 2019.^25^ Administered by the National Health Insurance Management Authority (NHIMA), the scheme aims to ensure equitable access to quality healthcare services for all Zambians, particularly the poor and vulnerable. Alongside NHIMA, several private health insurance providers also operate in the country,^26^ offering additional coverage options that further contribute to increased preventive service utilization. Together, these mechanisms form a key component of Zambia’s broader efforts to reduce financial barriers and advance progress toward Universal Health Coverage, which includes availability of cancer screening services.

Our study found that women who had visited a health facility in the past 12 months were significantly more likely to have undergone breast cancer screening. This finding is consistent with studies from other sub-Saharan African countries such as Lesotho,^27^ Kenya,^28^ and Ghana.^29,30^ In the absence of organized population-based screening programs, CBE may be provided during maternal health visits or other health consultations. This aligns with our finding that women who utilized cervical cancer screening services in Zambia were nearly six times more likely to have also received a breast examination. It reflects the Zambian Ministry of Health’s policy to integrate breast cancer screening services into existing platforms such as the Cervical Cancer Prevention Program and other Maternal and Child Health services.^11^ Leveraging existing infrastructure and trained personnel, this integrated service delivery model represents a cost-effective strategy to expand screening access and promote early detection, especially in low-resource settings.^31^

Additionally, we found that women living with HIV were less likely to have undergone breast screening in comparison to HIV-negative women. Evidence from Zambia suggests that this disparity may be partly driven by psychosocial factors, as women living with HIV may avoid cancer screening due to fear of social exclusion, terminal diagnosis, or marital breakdown.^32,33^ At the systemic level, this disparity may reflect the insufficient integration of breast cancer screening services within HIV care in the country. While HIV programs in Zambia have effectively expanded access to care and reduced AIDS-related mortality, they have not consistently incorporated non-communicable disease prevention services such as clinical breast exams into routine service delivery. This extends towards curative services, where women living with HIV in Zambia are reported to be less likely to receive curative treatment for breast cancer within the first 12 months of diagnosis as compared to their HIV-negative counterparts.^34^ Thus, there is a critical need to strengthen and scale up existing strategies such as integrated CBE and breast cancer education, which have been implemented in some clinics across the country.^31^

Geographical region was significantly associated with CBE uptake. In comparison to women residing in Lusaka, those in Central, Copperbelt, Luapula, and Northwestern Provinces were more likely to have undergone CBE, while those in Eastern Province were less likely. These variations likely reflect differences in health service accessibility and public health outreach across provinces. For example, Luapula and Northwestern Provinces have benefited from recent donor-supported outreach programs targeting cancer awareness, which may have increased screening exposure. Conversely, the lower uptake in Eastern Province could stem from limited availability of screening services, cultural barriers, and reduced awareness levels in more rural communities. These findings highlight the need to strengthen decentralized cancer screening programs and ensure equitable service distribution across provinces.

## Strengths and Weaknesses

This study has several weaknesses worth noting. First, the cross-sectional nature of the study limits the ability to infer causality. Second, the outcome i.e., breast examination by a provider, was measured by self-report, which could be subject to various biases such as recall and social desirability bias. Third, because the survey only covered women 15 through 49 years of age, our findings are not generalizable to older women. Future studies should include women above the age of 50. Additionally, important determinants such as family history of cancer and personal breast health knowledge were not measured, raising the possibility of residual confounding. Despite these limitations, the study strengths include its large, nationally representative sample, which enhance the generalizability of the results to Zambia’s population of reproductive age women. This research also fills a critical gap in context-specific CBE uptake in the country.

## Conclusion

There is low utilization of clinical breast cancer screening services among women aged 15–49 years in Zambia. Efforts that strengthen integrated service delivery ensuring that CBE is incorporated into existing health services and that minimize financial barriers through expanded health insurance coverage are warranted. At the same time, improving women’s health literacy is essential, as inadequate understanding of the benefits of early detection, fear of potential diagnoses or social consequences, and stigma, particularly among women living with HIV, may discourage screening even when services are available. Addressing these informational and psychosocial barriers alongside system level improvements is critical for increasing screening uptake and advancing equitable access to breast cancer prevention.

## Supplementary Material

Supplementary Files

This is a list of supplementary files associated with this preprint. Click to download.

- SupplementaryTable1.docx

## Figures and Tables

**Table 1:** Descriptive Statistics of reproductive aged women in Zambia

	Breasts examined for Breast Cancer			
Characteristic	N= 13 876 (100%)	No = 12 031 (86.7%)	Yes = 1845 (13.30%)	P-value
**Age group**				<0.001
15–19	3,290 (23.5)	3,131 (25.9)	159 (7.9)	
20–29	4,745 (34.4)	4,166 (34.9)	579 (31.3)	
30–39	3,476 (25.3)	2,835 (23.8)	641 (35.4)	
40–49	2,365 (16.8)	1,899 (15.4)	466 (25.5)	
**Type of residence**				<0.001
Urban	6,114 (51.0)	5,196 (50.1)	918 (56.5)	
Rural	7,762 (49.0)	6,835 (49.9)	927 (43.5)	
**Region**				<0.001
Central	1,623 (11.4)	1,362 (11.0)	261 (13.7)	
Copperbelt	1,563 (14.7)	1,293 (14.1)	270 (18.4)	
Eastern	1,519 (11.3)	1,401 (12.1)	118 (6.4)	
Luapula	1,261 (7.5)	1,038 (7.1)	223 (10.2)	
Lusaka	1,716 (18.8)	1,490 (18.8)	226 (18.6)	
Muchinga	1,012 (4.5)	893 (4.5)	119 (3.9)	
Northern	1,297 (7.5)	1,141 (7.6)	156 (6.7)	
Northwestern	1,165 (6.6)	1,029 (6.7)	136 (5.7)	
Southern	1,600 (11.7)	1,404 (11.9)	196 (10.9)	
Western	1,120 (6.0)	980 (6.1)	140 (5.6)	
**Highest educational level**				<0.001
No education	948 (6.4)	834 (6.6)	114 (5.8)	
Primary	5,808 (39.2)	5,111 (39.7)	697 (35.8)	
Secondary	6,166 (46.6)	5,389 (47.2)	777 (42.7)	
Higher	954 (7.8)	697 (6.5)	257 (15.8)	
**Wealth Status**				<0.001
Poorest	2,807 (17.4)	2,516 (18.0)	291 (13.5)	
Poorer	2,732 (17.1)	2,403 (17.4)	329 (15.4)	
Middle	2,821 (19.1)	2,454 (19.2)	367 (18.2)	
Richer	2,767 (22.4)	2,392 (22.4)	375 (22.3)	
Richest	2,749 (24.0)	2,266 (23.0)	483 (30.5)	
**Currently working**				<0.001
No	7,374 (53.1)	6,601 (54.9)	773 (41.7)	
Yes	6,502 (46.9)	5,430 (45.1)	1,072 (58.3)	
**Covered by health insurance**				<0.001
No	12,738 (90.6)	11,186 (91.9)	1,552 (82.2)	
Yes	1,138 (9.4)	845 (8.1)	293 (17.8)	
**Frequency of reading newspaper/magazine**				0.01
Not at all	12,014 (86.2)	10,481 (86.8)	1,533 (82.2)	
Less than once a week	699 (5.4)	561 (5.0)	138 (8.3)	
At least once a week	798 (5.8)	686 (5.8)	112 (6.3)	
Almost every day	365 (2.6)	303 (2.5)	62 (3.1)	
**Frequency of listening to radio**				0.02
Not at all	8,288 (58.3)	7,359 (59.8)	929 (48.6)	
Less than once a week	1,003 (8.1)	837 (7.8)	166 (10.1)	
At least once a week	2,444 (18.2)	2,062 (17.8)	382 (20.5)	
Almost every day	2,141 (15.4)	1,773 (14.6)	368 (20.7)	
**Frequency of watching television**				<0.001
Not at all	8,752 (58.8)	7,728 (59.9)	1,024 (51.8)	
Less than once a week	514 (3.9)	435 (3.9)	79 (4.4)	
At least once a week	1,280 (10.3)	1,078 (10.1)	202 (11.5)	
Almost every day	3,330 (27.0)	2,790 (26.2)	540 (32.4)	
**Frequency of using internet last month**				<0.001
Not at all	10,521 (72.2)	9,276 (73.5)	1,245 (63.8)	
Less than once a week	317 (2.5)	267 (2.5)	50 (2.6)	
At least once a week	949 (8.2)	821 (8.3)	128 (7.8)	
Almost every day	2,089 (17.0)	1,667 (15.7)	422 (25.8)	
**Current marital status**				<0.001
Never in union	4,598 (33.9)	4,270 (36.4)	328 (17.9)	
Married/Living with partner	7,424 (53.1)	6,226 (51.3)	1,198 (65.0)	
Widowed/Divorced/Separated	1,854 (13.0)	1,535 (12.3)	319 (17.1)	
**Age at menarche**				0.002
Early (<12)	506 (3.9)	431 (3.8)	75 (4.2)	
Normal (12–16)	12,024 (86.7)	10,460 (87.0)	1,564 (84.8)	
Delayed (>16)	1,098 (7.7)	909 (7.3)	189 (10.2)	
DK/Never menstruated	248 (1.7)	231 (1.9)	17 (0.8)	
**Sexual initiation**				<0.001
Never had sex	1,688 (12.7)	1,636 (14.2)	52 (2.8)	
Early (8–14)	2,382 (16.5)	2,061 (16.5)	321 (16.9)	
Normal (15+)	9,806 (70.7)	8,334 (69.3)	1,472 (80.2)	
**Parity**				0.02
0 children	3,692 (27.6)	3,470 (30.0)	222 (12.7)	
1 child	2,371 (17.0)	2,071 (17.1)	300 (16.0)	
2–4 children	4,837 (35.2)	4,036 (33.8)	801 (44.3)	
≥5 children	2,976 (20.2)	2,454 (19.1)	522 (26.9)	
**HIV result**				0.01
Positive	1,035 (9.3)	781 (8.3)	254 (14.7)	
Negative	10,476 (90.1)	8,965 (91.0)	1,511 (84.9)	
Indeterminate/No result	76 (0.6)	69 (0.7)	7 (0.4)	
**Self-reported health status**				0.18
Good	11,880 (85.4)	10,335 (85.8)	1,545 (83.0)	
Moderate	1,732 (12.6)	1,473 (12.3)	259 (14.6)	
Bad	264 (2.0)	223 (1.9)	41 (2.4)	
**Visited health facility in last 12 months**				<0.001
No	3,580 (26.6)	3,276 (28.1)	304 (16.8)	
Yes	10,296 (73.4)	8,755 (71.9)	1,541 (83.2)	
**Distance to health facility**				0.068
Big problem	4,218 (29.4)	3,756 (30.3)	462 (23.6)	
Not a big problem	9,658 (70.6)	8,275 (69.7)	1,383 (76.4)	
**Ever tested for cervical cancer**				<0.001
No	10,200 (72.7)	9,552 (78.8)	648 (33.8)	
Yes	3,676 (27.3)	2,479 (21.2)	1,197 (66.2)	

**Table 2:** Factors associated with clinical breast cancer screening among reproductive aged women in Zambia

	Crude Model		Multivariable Model	
Characteristic	cOR (95% CI)	p-value	aOR (95% CI)	p-value
**Age group**				
15–19	1 (Ref)	-	1 (Ref)	-
20–29	2.95 (2.41–3.63)	<0.001	1.05 (0.80–1.37)	0.72
30–39	4.89 (4.00–5.98)	<0.001	1.12 (0.83–1.52)	0.47
40–49	5.42 (4.41–6.68)	<0.001	1.22 (0.87–1.71)	0.25
**Type of residence**				
Rural	1 (Ref)	-	1 (Ref)	
Urban	0.77 (0.67–0.89)	0.0002	0.85 (0.69–1.04)	0.11
**Region**				
Lusaka	1 (Ref)	-	1 (Ref)	-
Central	1.26 (1.00–1.60)	0.053	1.43 (1.10–1.88)	0.01
Copperbelt	1.33 (1.05–1.67)	0.017	1.42 (1.12–1.78)	0.003
Eastern	0.53 (0.39–0.73)	<0.001	0.68 (0.49–0.95)	0.03
Luapula	1.45 (1.08–1.94)	0.013	1.94 (1.39–2.72)	<0.001
Muchinga	0.87 (0.62–1.22)	0.419	1.07 (0.73–1.56)	0.73
Northern	0.89 (0.66–1.19)	0.419	1.16 (0.86–1.56)	0.34
Northwestern	0.87 (0.66–1.14)	0.299	1.33 (1.01–1.77)	0.04
Southern	0.94 (0.73–1.20)	0.603	0.95 (0.72–1.27)	0.75
Western	0.92 (0.69–1.23)	0.574	0.81 (0.59–1.12)	0.21
**Highest educational level**				
No education	1 (Ref)	-	1 (Ref)	-
Primary	1.03 (0.82–1.28)	0.82	1.02 (0.79–1.32)	0.86
Secondary	1.03 (0.81–1.30)	0.811	1.00 (0.74–1.34)	0.99
Higher	2.74 (2.09–3.60)	<0.001	1.58 (1.08–2.32)	0.02
**Wealth Status**				
Poorest	1 (Ref)	-	1 (Ref)	-
Poorer	1.18 (0.97–1.43)	0.099	0.97 (0.79–1.20)	0.80
Middle	1.26 (1.04–1.53)	0.019	0.95 (0.76–1.18)	0.64
Richer	1.33 (1.09–1.61)	0.004	0.92 (0.69–1.23)	0.59
Richest	1.77 (1.45–2.14)	<0.001	1.02 (0.72–1.45)	0.89
**Currently working**				
No	1 (Ref)	-	1 (Ref)	-
Yes	1.70 (1.51–1.92)	<0.001	0.96 (0.84–1.11)	0.61
**Covered by health insurance**				
No	1 (Ref)	-	1 (Ref)	-
Yes	2.46 (2.10–2.88)	<0.001	1.30 (1.04–1.62)	0.02
**Frequency of reading newspaper/magazine**				
Not at all	1 (Ref)	-	1 (Ref)	-
Less than once a week	1.76 (1.43–2.17)	<0.001	1.58 (1.23–2.03)	<0.001
At least once a week	1.16 (0.92–1.47)	0.204	0.92 (0.70–1.21)	0.56
Almost every day	1.34 (0.98–1.83)	0.065	1.14 (0.79–1.65)	0.49
**Frequency of listening to radio**				
Not at all	1 (Ref)	-	1 (Ref)	-
Less than once a week	1.60 (1.31–1.97)	<0.001	1.20 (0.94–1.52)	0.14
At least once a week	1.42 (1.22–1.64)	<0.001	1.17 (0.99–1.37)	0.06
Almost every day	1.75 (1.51–2.03)	<0.001	1.18 (0.99–1.40)	0.06
**Frequency of watching television**				
Not at all	1 (Ref)	-	1 (Ref)	-
Less than once a week	1.33 (1.02–1.73)	0.033	1.14 (0.84–1.54)	0.39
At least once a week	1.31 (1.09–1.57)	0.004	1.14 (0.93–1.41)	0.21
Almost every day	1.43 (1.26–1.63)	<0.001	0.94 (0.77–1.15)	0.54
**Frequency of using internet last month**				
Not at all	1 (Ref)	-	1 (Ref)	-
Less than once a week	1.19 (0.84–1.69)	0.319	0.94 (0.63–1.41)	0.76
At least once a week	1.08 (0.86–1.35)	0.496	0.81 (0.62–1.05)	0.11
Almost every day	1.90 (1.67–2.17)	<0.001	1.16 (0.95–1.43)	0.15
**Current marital status**				
Never in union	1 (Ref)	-	1 (Ref)	-
Married/Living with partner	2.58 (2.24–2.97)	<0.001	1.22 (0.99–1.51)	0.06
Widowed/Divorced/Separated	2.82 (2.39–3.33)	<0.001	1.16 (0.92–1.47)	0.21
**Age at menarche**				
DK/Never menstruated	1 (Ref)	-	1 (Ref)	-
Early (<12)	2.44 (1.33–4.47)	0.004	1.38 (0.69–2.76)	0.37
Normal (12–16)	2.18 (1.29–3.66)	0.004	1.13 (0.62–2.06)	0.69
Delayed (>16)	3.12 (1.82–5.34)	<0.001	1.31 (0.71–2.41)	0.40
**Sexual initiation**				
Never had sex	1 (Ref)	-	1 (Ref)	-
Early (8–14)	5.17 (3.65–7.32)	<0.001	1.51 (0.89–2.58)	0.13
Normal (15+)	5.82 (4.21–8.05)	<0.001	1.43 (0.85–2.40)	0.18
**Parity**				
0 children	1 (Ref)	-	1 (Ref)	-
1 child	2.20 (1.82–2.67)	<0.001	0.93 (0.72–1.21)	0.60
2–4 children	3.08 (2.63–3.61)	<0.001	0.95 (0.72–1.25)	0.70
≥ 5 children	3.31 (2.78–3.94)	<0.001	1.11 (0.81–1.53)	0.50
**HIV result**				
Negative	1 (Ref)	-	1 (Ref)	-
Positive	1.90 (1.62–2.23)	<0.001	0.82 (0.69–0.98)	0.03
Indeterminate/No result	0.66 (0.29–1.49)	0.316	0.46 (0.19–1.13)	0.09
**Self-reported health status**				
Good	1 (Ref)	-	1 (Ref)	-
Moderate	1.22 (1.04–1.43)	0.012	0.93 (0.78–1.11)	0.42
Bad	1.33 (0.92–1.92)	0.129	1.13 (0.77–1.68)	0.53
**Visited health facility in last 12 months**				
No	1 (Ref)	-	1 (Ref)	-
Yes	1.94 (1.69–2.23)	<0.001	1.38 (1.18–1.61)	<0.001
**Distance to health facility**				
Big problem	1 (Ref)	-	1 (Ref)	-
Not a big problem	1.41 (1.22–1.62)	<0.001	1.17 (1.00–1.37)	0.06
**Ever tested for cervical cancer**				
No	1 (Ref)	-	1 (Ref)	-
Yes	7.25 (6.37–8.25)	<0.001	5.95 (5.18–6.84)	<0.001

## Data Availability

The data that support the findings of this study are available from the Demographic and Health Surveys Program. [https://dhsprogram.com/] (https:/dhsprogram.com)

## Abbreviations

AOR: Adjusted Odds Ratio
CBE: Clinical Breast Examination
CI: Confidence Interval
ZDHS: Zambia Demographic and Health Survey
